# Inkjet printing of NiO films and integration as hole transporting layers in polymer solar cells

**DOI:** 10.1038/s41598-017-01897-9

**Published:** 2017-05-11

**Authors:** Arjun Singh, Shailendra Kumar Gupta, Ashish Garg

**Affiliations:** Department of Materials Science and Engineering, Indian Institute of Technology Kanpur, Kanpur, 208016 India

## Abstract

Stability concerns of organic solar cell devices have led to the development of alternative hole transporting layers such as NiO which lead to superior device life times over conventional Poly(3,4-ethylenedioxythiophene) Polystyrene sulfonate (PEDOT:PSS) buffered solar cells. From the printability of such devices, it is imperative to be able to print NiO layers in the organic solar cell devices with normal architecture which has so far remained unreported. In this manuscript, we report on the successful ink-jet printing of very thin NiO thin films with controlled thickness and morphology and their integration in organic solar cell devices. The parameters that were found to strongly affect the formation of a thin yet continuous NiO film were substrate surface treatment, drop spacing, and substrate temperature during printing. The effect of these parameters was investigated through detailed morphological characterization using optical and atomic force microscopy and the results suggested that one can achieve a transmittance of ~89% for a ~18 nm thin NiO film with uniform structure and morphology, fabricated using a drop spacing of 50 μm and a heat treatment temperature of 400 °C. The devices fabricated with printed NiO hole transporting layers exhibit power conversion efficiencies comparable to the devices with spin coated NiO films.

## Introduction

Organic solar cells (OSC) are third generation photovoltaic devices which are being extensively researched due to their potential for flexible, low cost photovoltaic devices which could potentially be produced using manufacturing techniques such as printing technologies^1–3^. Most commonly used devices are based on bulk heterojunction configuration in which the main light absorbing layer is made of a blend of a p-type polymer such as Poly(3-hexylthiophene-2,5-diyl)(P3HT) and a n-type acceptor, typically a fullerene such as Phenyl-C61-butyric acid methyl ester (PC_61_BM) with devices yielding efficiencies of the order of 3–4%. More recently, advent of new blend systems such as Poly{2,6-bis(3-dodecylthiophen-2-yl) benzo[1,2-b;4,5-b’]dithiophene}(PTBT)^4^, Poly[4,8-bis(5-(2-ethylhexyl) thiophen-2-yl) benzo[1,2-b;4,5-b’]dithiophene-2,6-diyl-alt-(4-(2-ethylhexyl)-3-fluorothieno[3,4-b]thiophene-)-2-carboxylate-2-6-diyl)] (PTB7-Th)^5^, Poly(dithieno[3,2-b:2,3-d]germole thieno[3,4-c]pyrrole-4,6-dione)(PDTG–TPD)^6, 7^, Poly{2,6′-4,8-di(5-ethylhexylthienyl) benzo[1,2-b;3,4-b]dithiophenealt-2,5-bis(2-butyloctyl)-3,6-bis(selenophene-2-yl) pyrrolo[3,4-c]pyrrole-1,4-dione} (PBDTT-SeDPP)^6^, Poly[[4,8-bis[(2-ethylhexyl) oxy]benzo[1,2-b:4,5-b’]dithiophene-2,6-diyl][3-fluoro-2-[(2-ethylhexyl) carbonyl]thieno[3,4-b]thiophenediyl]](PTB7)^8^ and [6,6]-phenyl-C_71_-butyric acidmethyl ester (PC_71_BM) have led to efficiencies over 7–8% marking a promising future for OSCs. The blend layer in an OSC device is sandwiched between the two electrodes, typically a transparent oxide such as indium tin oxide (ITO) and a metal (e.g. Al) along with the presence of buffer layers which facilitate the flow of carriers to respective electrodes. A typical configuration is Glass/PEDOT:PSS/P3HT:PC_61_BM/Ca/Al where PEDOT:PSS is used as a hole transport layer. However, the devices using PEDOT:PSS have very short life times due to its corrosive nature and hence present a bottleneck to any commercial realization of these devices.

As a result, other alternatives hole transporting materials have been incorporated into OSC devices with the primary aim of improving the device life times whilst not sacrificing the device efficiencies. Among these, transition metal such as NiO^9^, MoO_3_^10^ and WO_3_^11^ appear to have substantial promise as use of these as hole transporting layer results in substantial improvement in the device life times. Whilst NiO and MoO_3_ buffered P3HT:PC_61_BM devices show comparable device efficiencies compared to PEDOT:PSS buffered devices, WO_3_ buffered devices show lower device efficiencies. The differences arise because the performance of an organic solar cell device is strongly affected by various attributes of these interlayers, for example the charge collection efficiency of the interlayer depends on its work function^12–15^ and conductivity^16^ as well as energy level alignment of the interlayer with the lowest occupied molecular orbital (LUMO) and highest occupied molecular orbital (HOMO) of the materials across the heterojunction^17–21^.

Among these candidates, NiO is a good choice because it is a very stable oxide, is a known p-type material and NiO thin films can be deposited using both physical vapour deposition^22^, electrodeposition^23, 24^, chemical bath deposition^25^ as well as spray pyrolysis^26^ methods. Irwin *et al*.^9^ first used pulsed laser deposition (PLD) to deposit NiO thin films as hole transporting layer for integration with P3HT:PCBM blend based solar cells^9, 27^ with devices showing comparable efficiencies with much improved stability over a period of 21 days^9, 27, 28^. Further, Olson *et al*. demonstrated solution processed NiO films as hole transport layers^29^ and obtained OSC device efficiency ~3.6%, similar to control PEDOT:PSS device. The spin coated NiO based devices also showed lower series resistance as compared to control PEDOT:PSS. In other works, in conjunction with P3HT:PC_61_BM blend, the NiO buffered devices show efficiencies exceeding 4–5%^30, 31^. Subsequently, Jesse *et al*.^32^ achieved 7.8% efficiency with NiO as HTL in conjunction with blend of pDTG-TPD and PC_71_BM as active layer. A key parameter that affects the performance of NiO films in OPV devices is the annealing temperature of NiO film^31^. A few reports also showed the improvement in the device performance with surface treatment of NiO film using oxygen plasma, UV ozone which was attributed to the change in the work function of NiO^33–35^. Solution processed NiO films have also been used as hole-extracting layers in perovskite solar cells^36–38^ with cell efficiencies up to 14.0%.

Hence, given the importance of NiO for OSC devices with improved life times, it is vital to be able print NiO films from the perspective of large area device development. Although a few reports have demonstrated printing of NiO films, all have been to fabricate thick NiO films, for example screen printed thick NiO films for gamma radiation detection applications^39^, thick NiO electrodes by ink-jet printing^40^, and printing of NiO nanoparticles for thermistor^41^ and electrochromic applications^42^. To the best of our knowledge, there are no studies on printed NiO thin films for organic solar cell devices. Particularly critical is the control of thickness at thicknesses as low as 20–30 nm which makes it a challenging task as one requires control of both uniformity and morphology and hence a detailed understanding of the process is required.

This articles demonstrates that one can successfully print thin, uniform and smooth NiO films using ink-jet printing with control of various process parameters which can be integrated with organic solar cells. The workclearly shows the details of fabricating a solution-processed inkjet printed NiO thin layer as a hole transparent layer (HTL) followed by its integration with P3HT:PC_60_BM based bulk heterojunction junction solar cells demonstrating good device performance when compared to the control samples fabricated using spin coated NiO thin films. The stability testing of the device consisting of printed NiO hole transporting layers showed significantly better performance over devices containing conventional PEDOT:PSS hole transporting layers.

## Results and Discussion

### Effect of surface treatment on printing of NiO films

**Figure 1:**
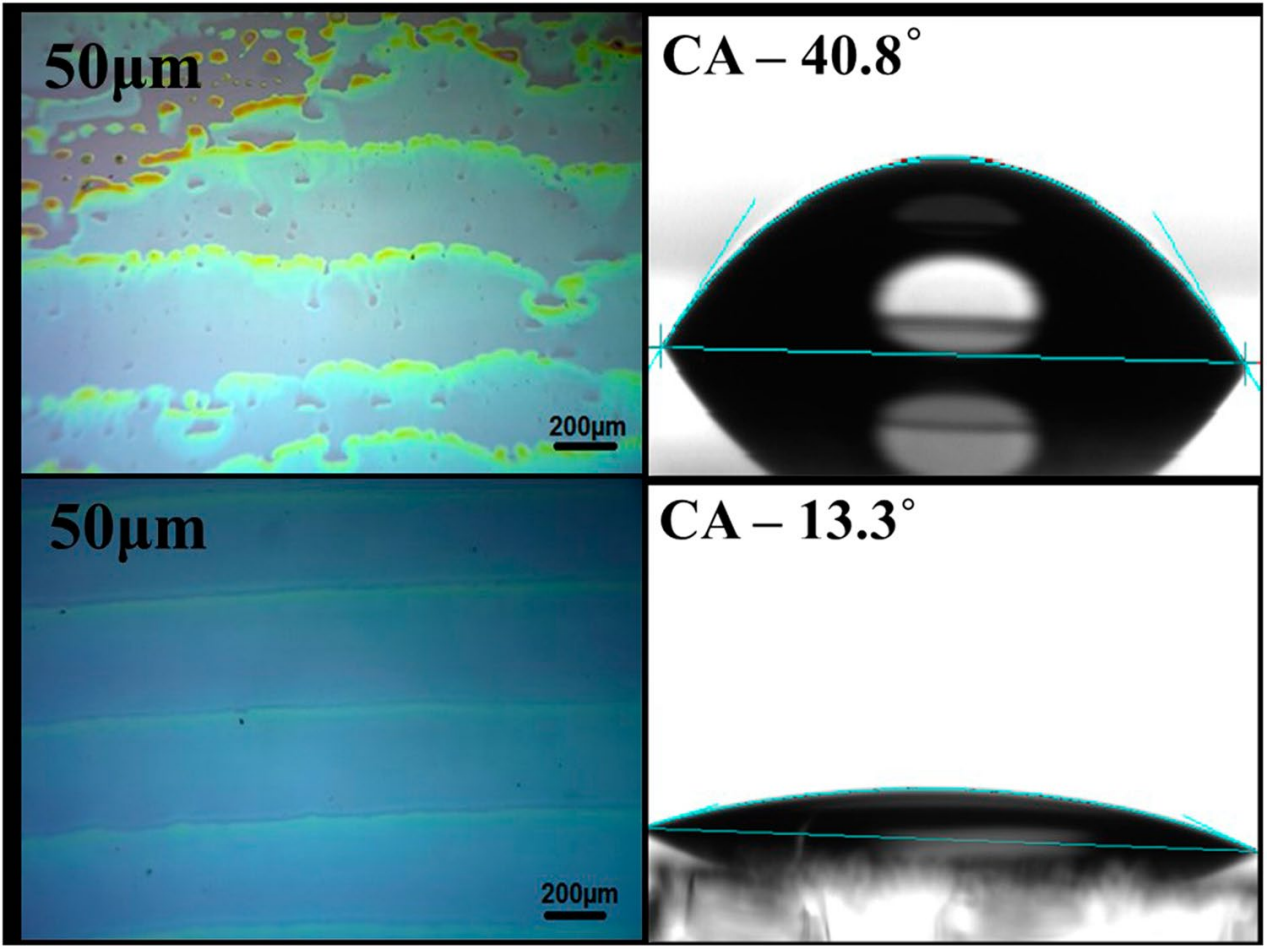
Left: Optical micrographs of printed NiO films printed at a drop spacing ~50 μm, Right: Contact angle measurement images on ITO/Glass substrates with and without UV-Ozone treatment.

Based on the approach adopted in ink-jet printing of PEDOT:PSS and ZnO^43, 44^, spherical drops of NiO precursor ink were formed upon printing at a jetting voltage of 12V using ink of surface tension 36.88 dynes/cm, viscosity 2.6 cP (see supplementary information). The corresponding value of dimensionless parameter Fromm Number (Z)^45^ was 11.4. There is no consensus in the literature over which value of Z yields a stable drop with a few reports indicating that a spherical droplet form without any satellite drops when Z is greater than 2^45^ and while a few other reporting that the value 4 ≤ Z ≤ 14^46^ is appropriate for stable droplet formation. Using above printing parameters and 50 µm as drop spacing, we studied the ink spreading behavior on surface treated(UV-Ozone treated) and untreated ITO coated glasses substrate and results are shown in Fig. 1. As observed from Fig. 1, although the ink spreads on both the substrates, the resulting film on UVO treated substrate is far more uniform than the one on untreated substrate indicating non-uniform spreading of ink on the ITO substrates having no UV-Ozone treatment. This results was further confirmed by measurement of water contact angle on treated and non-treated surfaces (Fig. 1). The measurements showed that UV-Ozone treatment of the ITO substrate surface leads to a decrease in the contact angle of NiO drop on the substrate surface from 40.8° on untreated surface to 13.3° on UVO treated surface. Hence, for all the subsequent studies, we used UVO treated substrates.

### Effect of variation in drop spacing

**Figure 2:**
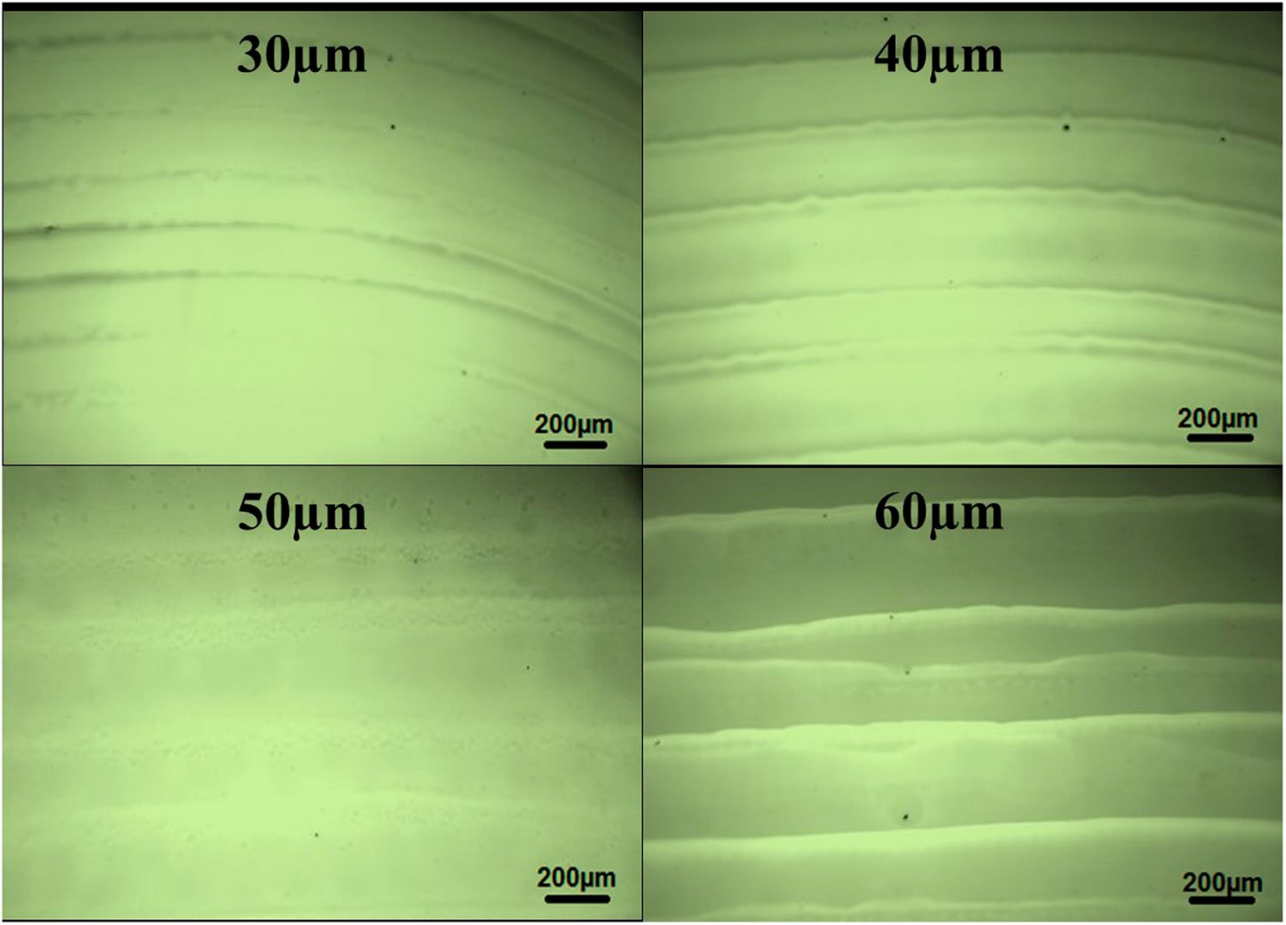
Optical images of inkjet printed NiO films at different drop spacing (30, 40, 50 and 60 μm) on UV-Ozone treated ITO/Glass substrates.

Next, we studied the drop overlapping (or drop spacing) effect on the uniformity of inkjet printed films on the ITO substrate printed at room temperature (RT) i.e. 25 °C. The results as shown in Fig. 2 show that the surface of the film is most uniform or flat at a drop spacing of 50 μm and becomes non-uniform as the drop spacing is decreased or increased. With increase in the drop spacing, both the width of the line as well as the edge height decrease as observed from the line profiles of individually printed lines (see supplementary information). At lower drop spacing, the distance between the two adjacent drops is smaller resulting in more volume of ink along a line per unit length and as the drop spacing increases, the ink volume per unit length reduces. As a result, the overlapping of drops has to be just optimum for a smooth film as higher overlapping will result in a thicker as well as non-uniform film while lesser overlapping will result in a discontinuous film. Hence for all the further experiments, we chose a drop spacing of 50 μm.

### Effect of substrates temperature on printing

**Figure 3:**
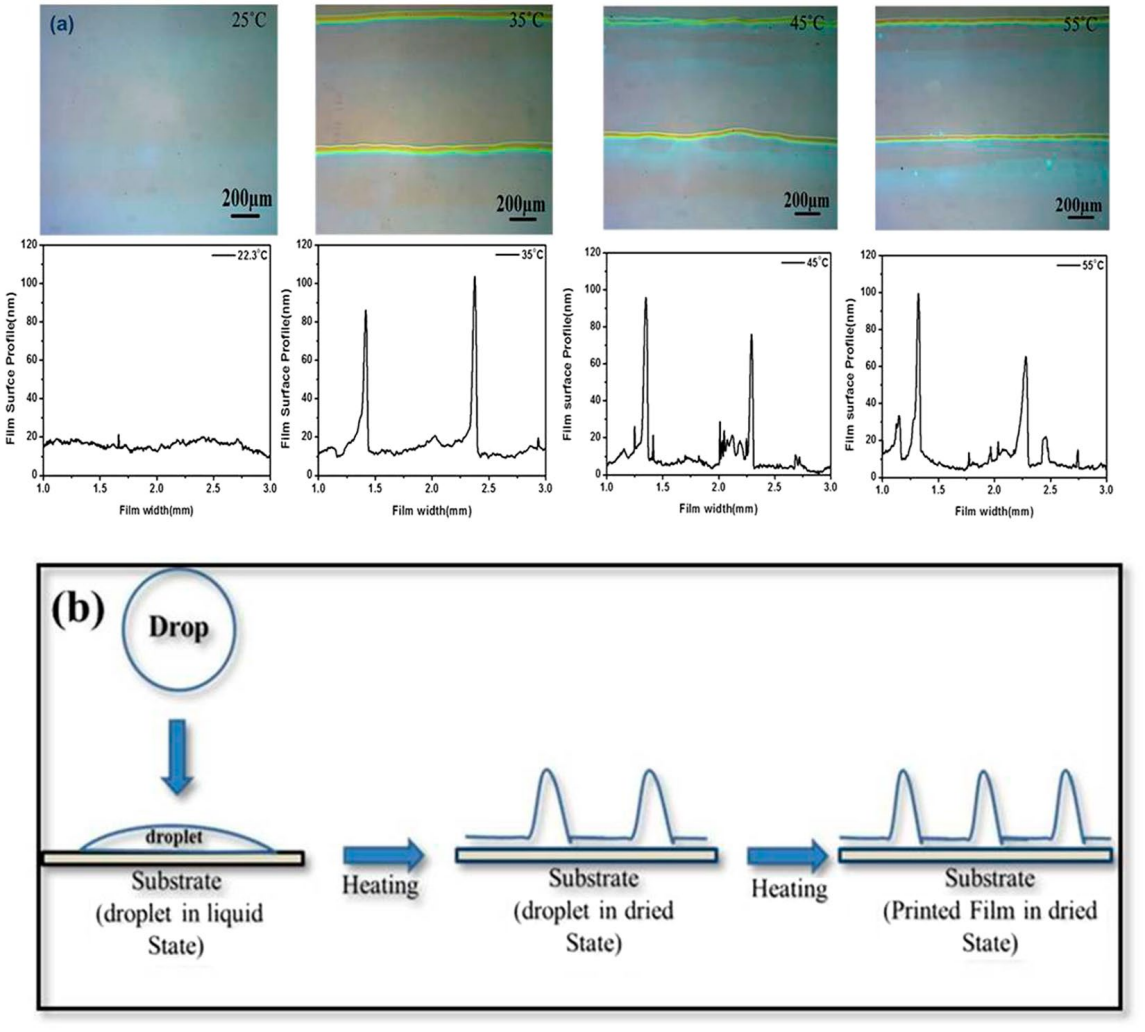
(**a**) Optical images and 2D profiles of printed NiO films at various substrates temperature on ITO/glass substrates with UVO surface treatment, keeping the drop spacing at 50 μm. (**b**) Schematic diagram of drying behavior of NiO film at higher substrate temperatures during printing.

Further we checked the effect of substrate temperature on the quality of the surface of printed NiO films. The NiO precursor ink was printed on UV-Ozone treated substrates at various substrates temperatures i.e. 25, 35, 45 and 55 °C followed by annealing at 400 °C for 1 hour in the ambient. As shown in Fig. 3(a), increasing the substrate temperature leads to an increased non-uniformity of the films, depicted by wavy profiles of the films deposited at the substrate temperatures at and above 35 °C. Also, the number of lines as well height of lines on the printed film increases with increase in the substrate temperature, similar to that observed in Fig. 2. This again illustrates that the increasing the substrate temperature beyond RT restricts the spreading of ink resulting in failure of printed lines to merge due to faster evaporation of the solvent and in none of the printed films at temperatures at and above 35 °C, lines showed a tendency to merge and form continuous film. The printed area in these films was 1.5 × 1.5 cm^2^, which allows one to achieve a large enough uniform area avoiding the edge effects. Based on this, NiO ink was printed on UV-Ozone treated ITO/Glass substrates at RT with drop spacing of 50 μm having a film thickness 18 nm. The evaporation rate of solvent increases with increase in the substrate temperature during printing of NiO inkand that causes the movement of solvent to the edges as shown in the schematic diagram (Fig. 3(b)), affecting the uniform spreading of the ink. This uneven spreading of ink is manifested in a non-uniform NiO film surface as shown in the Fig. 3(a). In the same manner, due to faster evpoartion of solvent, the width of the lines printed on the substrates also reduces with increase in the substrate temperature during printing of NiO ink, as can be seen in supplementary information.

### Annealing temperature effect on structure and optical properties of inkjet printed NiO films

**Figure 4:**
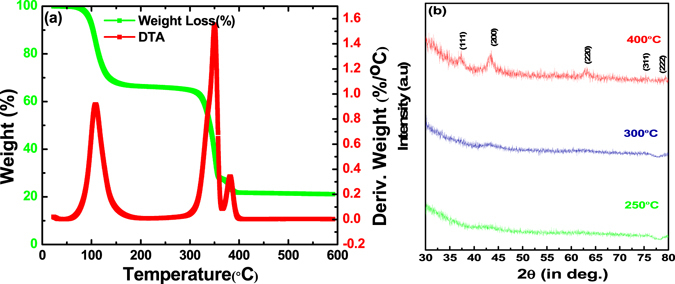
(**a**) Thermogravimetric (TGA) analysis of nickel oxide precursor ink used for printing of NiO thin films and (**b**) XRD spectra of printed NiO films on glass substrates heat treated at different temperatures for one hour.

To study the effect of heat treatment on the printed NiO films and subsequent effect on the devices, we first subjected the NiO ink consisting of nickel acetate tetrahydrate as a precursor to thermogravimetric analysis (TGA) from room temperature to 600 °C in nitrogen ambient. The results, as shown in Fig. 4(a), suggest that there are two major weight loss regimes: 35% loss in the vicinity of 100 °C and of about 45% near 350 °C, as depicted by sharp peaks in the differential plot. The first regime corresponds to the vaporization of water, loosely bound with the precursor or present in the form of moisture. The second peak at ~350 °C corresponds to the loss of water through decomposition of the precursor leading to nickel oxide formation^47–50^ which can be represented by the following reaction^51^:

1 $${\rm{Ni}}{({{\rm{CH}}}_{3}{\rm{CO}})}_{2}.4{{\rm{H}}}_{2}{\rm{O}}+2{{\rm{CH}}}_{3}{\rm{OH}}\to {\rm{Ni}}{({\rm{OH}})}_{2}+2{{\rm{CH}}}_{3}{{\rm{COOCH}}}_{3}+4{{\rm{H}}}_{{\rm{2}}}{\rm{O}}$$

2 $${\rm{Ni}}{({\rm{OH}})}_{{\rm{2}}}+{{\rm{2CH}}}_{{\rm{3}}}{{\rm{COOCH}}}_{{\rm{3}}}\to {\rm{NiO}}+{{\rm{H}}}_{{\rm{2}}}{\rm{O}}$$

Based on these results, we chose three heat treatment temperatures: 250 °C (above the first water loss regime), 300 °C (below second weight loss regime)) and 400 °C (above the second weight loss regime beyond which no weight change occurs).

**Figure 5:**
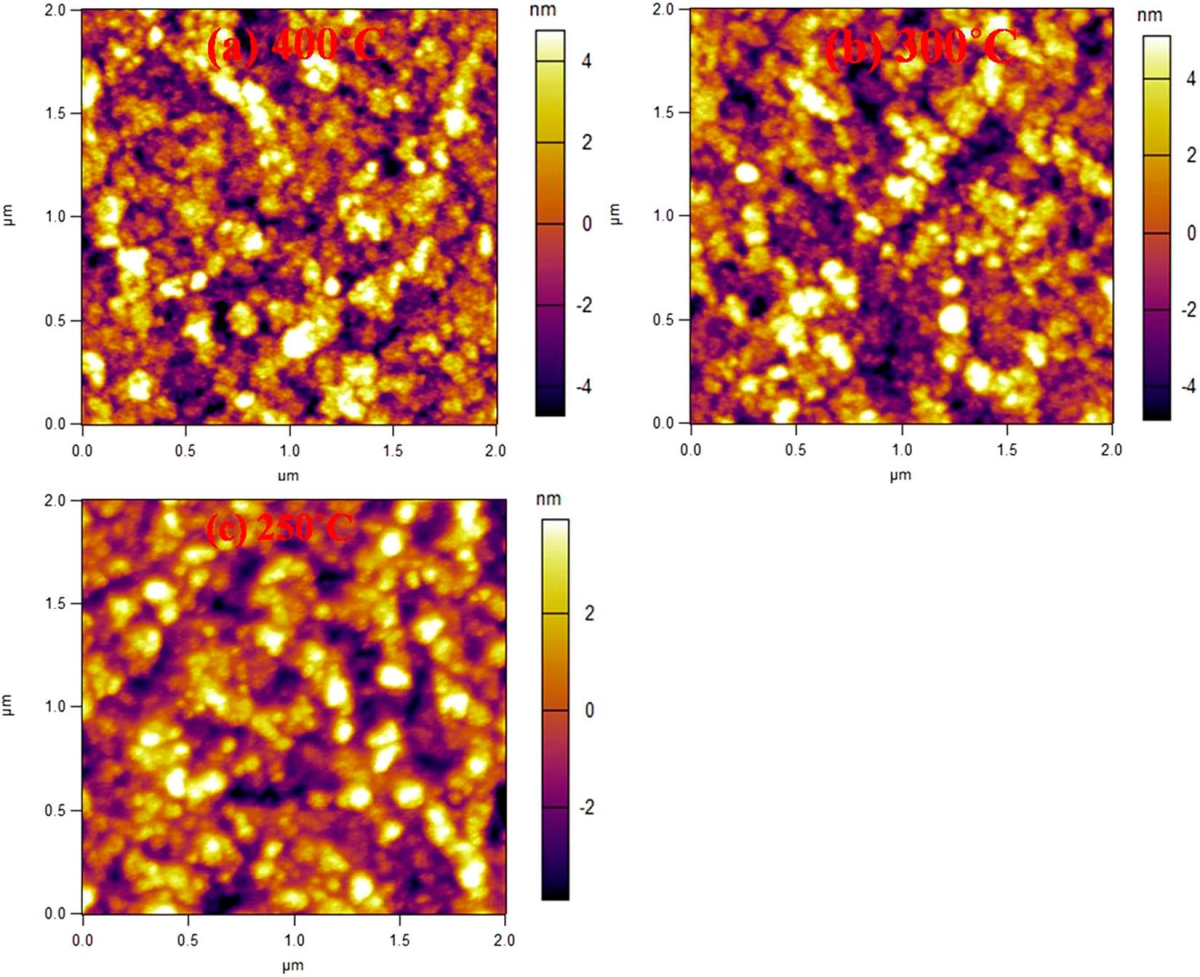
AFM images of NiO films printed on ITO coated glass heat treated at (**a**) 400 °C (**b**) 300 °C (**c**) 250 °C.

Further, 18 nm thin NiO films were printed (drop spacing ~50 µm) on ITO coated glass substrates held at room temperature followed by heat treating at 250, 300 and 400 °C in air for one hour and the corresponding X-ray diffraction spectra of the samples are shown in Fig. 4(b). The spectra clearly show that while the films heat treated at 250 and 300 °C are amorphous without presence of any peaks, the film heat treated at 400 °C is possibly nanocrystalline as evident from the presence of rather broad yet characteristics diffraction peaks. The average crystallite size of the NiO film annealed at 400 °C was 6.2 ± 1.8 nm confirming the nanocrystallinity, calculated from the X-ray line broadening using Scherrer formula $D=\frac{0.94\lambda }{\beta cos\theta }\,$after subtracting the instrumental broadening (D: crystallite size, θ:Bragg position, **β**: full width at half maxima(FWHM), λ: wavelength of CuKα radiation). However, since the broadening also consists of thin film strain, which could not be ascertained due to weak peaks, the crystallite size is likely to be moderately higher. Further morphological analysis conducted using AFM, as shown in Fig. 5(a–c), suggests that the film are uniform with surface roughness of 2.32 ± 0.14 nm (400 °C), 2.20 ± 0.28 nm (300 °C) and 1.75 ± 0.05 nm (250 °C) with the films showing crystallite size of below 50 nm in all the films.

**Figure 6:**
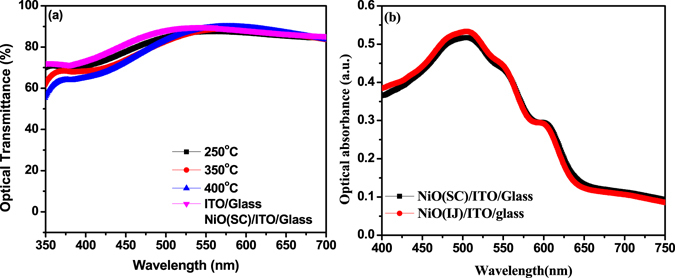
(**a**) Transmission spectra of printed NiO films and spin coated NiO films (as reference) and (**b**) UV-Vis absorbance spectra of 70 nm thin P3HT:PC_60_BM films on printed NiO and spin coated NiO films on ITO/Glass substrates.

Optical transmission characteristics of the heat-treated 18 nm thin NiO films are shown in Fig. 6 with results of spin coated 10 nm thin NiO film on ITO coated glass substrates and bare ITO coated glass substrates shown as reference. The results suggest that there are not substantial differences between the transmission characteristics of spin coated NiO films and inkjet printed films with spin coated films showing slightly better transmission above ca. 540 nm while printed NiO films are optically slightly more transparent in the region above 520–550 nm with maximum transmittance at ~89% at 590 nm of the films heat treated at 400 °C. NiO films heat-treated at 400 °C are slightly more transparent than those heat-treated at 300 and 250 °C which could perhaps be attributed to slight crystallization of the film and thus leading to lower density of mid-gap states. However, this may require a deeper investigation as relation between amorphous structure and transparency can vary depending on the material.

Next, we measured the optical absorbance of P3HT:PC_60_BM (70 nm) as active layer which was spin coated on 18 nm thin printed NiO film (heat treated at 400 °C) as well as 10 nm thin spin coated NiO films on ITO/Glass substrates. The UV-Vis absorption spectra of these samples, as shown in Fig. 6(b), show that both the samples show nearly comparable absorption all through the measurement range with minor differences within the experimental error.

### Integration of printed NiO films in organic solar cell (OSC) devices

**Figure 7:**
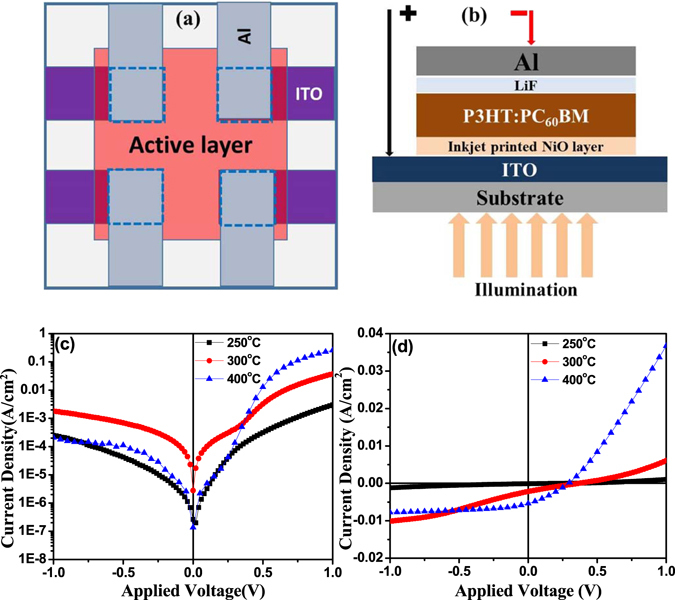
(**a**) The geometry of the electrode and (**b**) configuration of the OSC device (dotted square represent the device area(3 × 3mm^2^) while the glass substrate was of size 1.0 inch^2^); (**c**) Dark and (**d**) light J-V characteristics of P3HT:PC_60_BM organic solar cell devices fabricated on inkjet printed NiO hole transport layers, heat treated at various temperatures.

P3HT:PC_60_BM organic solar cells were fabricated on inkjet printed NiO thin films heat treated at various temperatures with device structure ITO/NiO(IJ)/P3HT:PC_60_BM(70 nm) (/LiF (1 nm)/Al. For comparison devices were also made on spin coated NiO films. The schematic of electrode geometry as well as device configuration are shown in Fig. 7(a,b). We measured the device characteristics of these samples under illumination and the results are discussed in the subsequent sections.

### Effect of annealing temperature of NiO film on the device performance

Figure 7(c,d) show the current density (J) vs voltage (V) characteristics of the devices measured under dark and light conditions. A comparison of dark J-V characteristics (Fig. 7(c)) of the OSC devices on printed NiO films heat treated at various annealing temperatures reveals that good diode like device characteristicsare obtained on NiO films heat treated at 400 °C with highest rectification ratio (I_ON_:I_OFF_). Same is also reflected in the J-V characteristics under illumination (Fig. 7(d)) with devices on NiO films heat treated at 400 °C showing the best J-V curves. Whilst the open circuit voltage remains at ~0.3 V for all the three devices, the fill factor and the J_SC_ increase substantially with increase in the heat treatment temperature of NiO films with highest J_SC_ of 5.4 mA/cm^2^ and device efficiency of ca. 0.7% achieved for the device on NiO film heat treated at 400 °C. Dramatic change in the J-V characteristics of NiO films heat treated at 400 °C is attributed to crystallization of the NiO film after complete thermal decomposition of nickel acetate tetrahydrate, used as a precursor to prepare the ink as indicated by TGA plots shown in Fig. 4(a)). However, in comparison to standard P3HT:PC_60_BM devices which regularly show efficiencies above 3%, the device performance is still quite poor as depicted by much lower J_SC_, V_OC_ and fill factor values, needing further process development. What is clear though is that higher temperature heat treatment leads to a higher photoconductivity of the active layer as shown by higher short circuit current, in agreement with the previous reports using spin coated NiO films using different precursor material^52^.

One concern with high temperature treatment is that it can lead to degradation of ITO as literature reports mention that ITO tends to degrade upon high temperature heat treatment due to Indium (In) diffusion leading to increase in the resistance^53^. However, our results show that annealing at 400 °C improves the device performance than annealing at 300 °C. Prima-facie our results do not suggest any degradation of the electrode as if that was the case, we should not have observed improved V_OC_ and the efficiency. In any case, it would be worth examining in a separate study as to why does ITO degradation not occur at 400 °C with NiO on it ? Could it be because NiO, having a closed packed structure, acts as a diffusion barrier protecting ITO?

### Surface treatment studies of NiO film and effect on device performance

**Figure 8:**
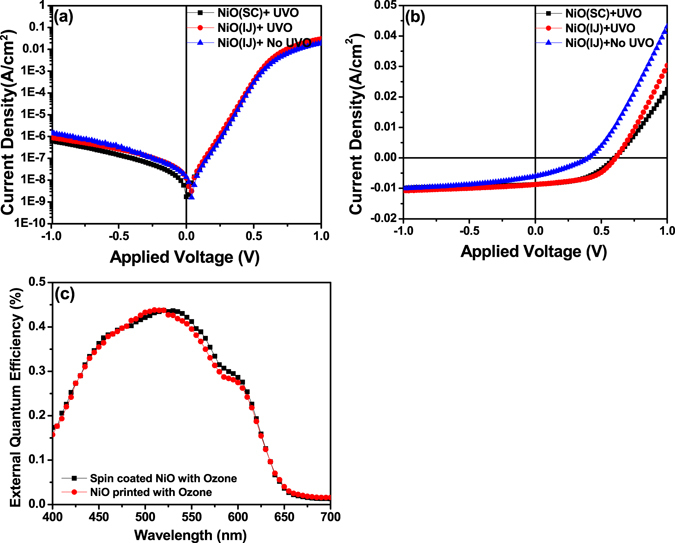
(**a**) Dark and (**b**) lightJ-V characteristics of P3HT:PC_60_BM organic solar cell devices fabricated on printed and spin coated NiO films heat treated at 400 °C (with and without UV-Ozone treatment) and (**c**) EQE spectra of the devices fabricated on spin coated and printed NiO films heat treated at 400 °C.

A key factor that affects the performance of NiO thin films is the surface treatment of NiO films prior to the deposition of active layer, which is shown to result in efficiency improvement of organic solar cell devices^30, 31^. To study this, we fabricated the OSC devices on printed NiO films which were heat treated at 400 °C for 1 h in ambient conditions and were UVO treated for 15 minutes immediately prior to the deposition of active layer. For comparison, devices were also fabricated on spin coated NiO films which were also UVO treated for 15 min and were heat treated at 400 °C prior to device fabrication. The dark and light J-V characteristics of these devices are shown in Fig. 8(a,b) and are also summarized in Table 1. While there is very marginal difference in the dark J-V characteristics of all the devices for both kinds of NiO films, there is substantial improvement in the photovoltaic performance under illumination from 0.75% for the device on NiO film without any surface treatment to 2.67% for the device on NiO film with 15 minutes UVO treatment, accompanied by the improvement in the V_OC_, J_SC_ and FF values. As the data in Table 1 shows, after UVO treatment, the sheet resistance of device on the UVO treated NiO film drops to half of the value obtained for device on untreated NiO films. Further, as Fig. 8 and Table 1 show, the performance of devices on printed and treated NiO is slightly better than spin coated NiO films as evident from slightly higher FF and J_SC_, also supported by nearly identical EQE spectra of the two devices (Fig. 8(c)). This clearly illustrates that inkjet printing process can result in the thin NiO films of comparable or even better quality than spin coated films resulting in good quality organic solar cell devices. UVO treatment of NiO films leads to lower series resistance and higher shunt resistance of the devices which is indicative of improved carrier transport, less electrical leakage as well as lesser recombination of charge carriers in these devices.

**Table 1:** Device parameters of P3HT:PC_60_BM organic solar cell devices fabricated on inkjet printed as well as spin coated NiO films used as HTL on ITO/Glass substrate, both heat treated at 400 °C and surface treated prior to device fabrication (UVO: UV Ozone, IJ: ink-jet printed, SC: spin coated).

Sample	NiO(IJ) + UVO	NiO(IJ) + No UVO	NiO(SC) + UVO
NiO Thickness	18 nm(IJ)	18 nm(IJ)	20 nm
V_OC_ (V)	0.60 ± 0.01	0.4	0.60
J_SC_ (mA/cm^2^)	8.57 ± 0.13	6.06 ± 0.04	8.42 ± 0.23
FF (%)	50.1 ± 0.76	31.22 ± 0.16	45.26 ± 1.86
PCE (%)	2.59 ± 0.08	0.75	2.28 ± 0.14
R_S_(Ω-cm^2^)	18	32	23
R_sh_(Ω-cm^2^)	311	123	461

**Figure 9:**
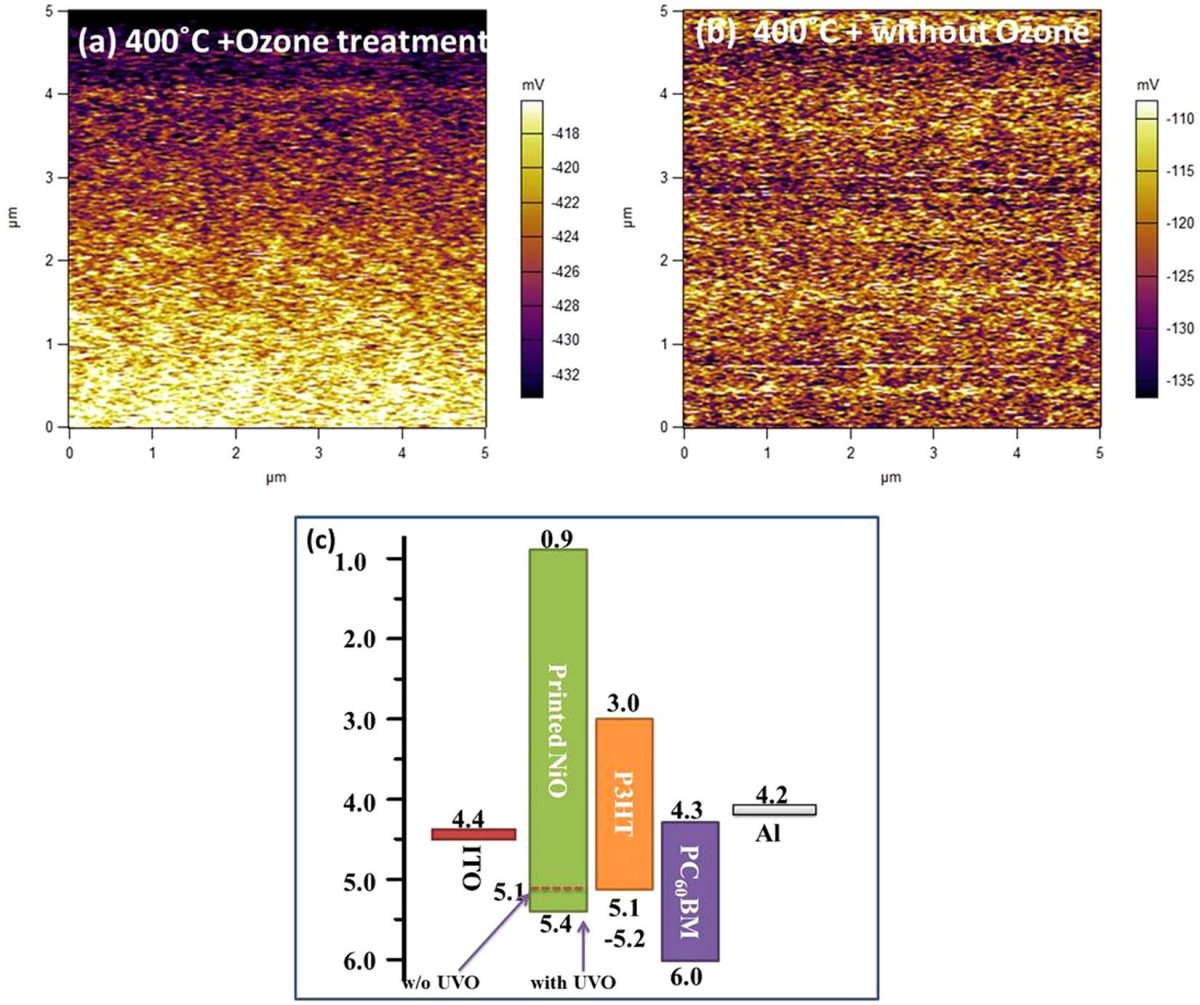
(**a**) and (**b**) KPFM contact potential difference maps of heat treated NiO films on ITO/glass substrates with and without ozone treatment of NiO film (**c**) Schematic energy band diagram of the device with NiO as HTL and P3HT:PC_60_BM active layer.

It has been suggested in the literature that the changes in the device performance after surface treatment of NiO films are likely to be due to oxidation of surface of printed NiO films after UV ozone treatment. It has been suggested that as annealed NiO surface is slightly reduced i.e. is of the form NiO_1−x_ and oxygen plasma treatment leads to further oxidation of metallic surface states. The deep work function of such oxidized interface with the active layer allows for the efficient extraction of holes due to a reduced energy barrier in comparison to that of the as annealed but untreated NiO film^30^. Oxidation of the surface layers of NiO films and adsorption of moisture leads to the formation of NiOOH species on the surface which forms surface dipoles, resulting in a change in the work function of the NiO film leading to improvement in the device performance as suggested by ultraviolet photoelectron spectroscopy or UPS measurements^31, 54^. To investigate whether the improvement in the device performance after UVO surface treatment of inkjet printed NiO film heat treated at 400 °C is indeed related to surface potential changes, we examined the surface potential of heat treated NiO films (with and without UVO surface treatment) using Kelvin probe force microscopy (KPFM). KPFM is an atomic force microscope based technique, which allows one to determine the changes in the work function and the surface potential in a non-destructive manner^55^. Although measurements in vacuum are more reliable, comparative measurements in air can also be useful to determine the surface energetics. In KPFM, the distributed contact potential difference (V_CPD_) between the scanning tip and the sample is calculated as ${{\boldsymbol{V}}}_{{\boldsymbol{CPD}}}=\frac{{{\rm{\varphi }}}_{{\boldsymbol{tip}}}-{{\rm{\varphi }}}_{{\boldsymbol{sample}}}}{{\boldsymbol{e}}}$ where φ_tip_(~ 5.0 eV) and φ_sample_ are the work functions of the tip and sample respectively and e is the electronic charge. Here, φ_tip_ has higher work function and a negative external voltage has been employed to the sample to nullify the V_CPD_. The KPFM images, as shown in Fig. 9(a,b), depict the surface potential maps of the surface of two printed NiO films, heat treated at 400 °C, with and without the surface treatment. As the scale bar of the images shows, the contact potential difference (CPD) values of two samples are: −424.75 mV (with UVO treatment) and −122.32 mV (without ozone treatment). The work function of NiO surface as estimated from these measurements is: 5.40 eV (with UVO treatment) and 5.12 eV (without the surface treatment) clearly showing that the work function of NiO increases with after the UVO surface treatment of printed NiO film and this increase is also reported in literature^29, 32, 34, 56^.

However, the increase in the work function of NiO alone cannot explain the efficiency improvement as it actually going to lead to a slightly increased interfacial barrier for holes travelling from P3HT to NiO, as shown in Fig. 9(c), although interface barrier is within the range required for making an Ohmic contact. Hence, the reason why UVO surface treatment of NiO film leads to improved device performance could lie in the improved interface quality between UVO treated NiO and the P3HT:PC_60_BM which leads to reduced trap density causing less recombination, and hence an improved V_OC_ from 0.4 V (without surface treatment) to 0.60 V (with UVO treatment) and an improved fill factor FF from 31% to 50%. To understand the effect of NiO surface treatment on the device performance, the built-in voltage (V_bi_) of the devices was calculated from the dark J-V data using the function $\frac{d\,\mathrm{log}\,j}{d\,\mathrm{log}\,V}$ as described in the approach used in ref. 57. The results show that V_bi_ increases from 0.71 V for untreated NiO film to 0.86 V for UVO treated NiO film and is also consistent with the enhancement in V_OC_. Increased V_bi_ suggests towards improved carrier extraction at ITO anode due to decrease in the surface trap density at the interface between the UVO treated NiO and the active layer, also evident from the improved shunt resistance signifying reduced shunt leakage. Moreover, the decreased series resistance of the device of UVO treated NiO also signifies improved conductivity of the active layer resulting in improved short circuit current density.

**Figure 10:**
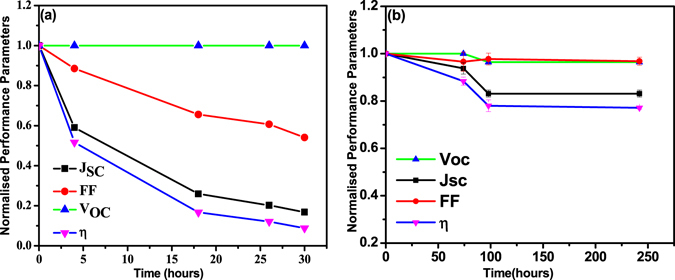
Stability of OPV devices with (**a**) PEDOT:PSS and (**b**) printed NiO as hole transport layers.

Finally, we examined the stability of printed NiO buffer layer based OPV devices. For comparison, we also tested the control devices fabricated on PEDOT:PSS coated substrates and the results are shown in Fig. 10. These devices were stored without any encapsulation in the ambient conditions (in dark, 25 °C and 40–45% relative humidity). While printed NiO based devices showed initial degradation of ca. 10% in the power conversion efficiency storage for 72 hours and ca. 23% after 120 hours before stabilizing when tested up to 250 hrs. The initial degradation of the devices could be attributed to the instability of LiF/Al interface perhaps due to Li diffusion towards the cathode interface affecting the device performance^58^. However, since, LiF is a very thin layer, its impact was limited. In comparison, the devices fabricated on PEDOT:PSS coated substrates showed rapid degradation within first few hours itself, a well known phenomenon which is attributed to corrosiveness of PEDOT:PSS which affects both the bottom electrode and the active layer adversely. The comparison of two results clearly suggests the beneficial role of NiO layer on device stability. It would be of further interest to examine the stability aspects for longer times and under accelerated conditions of temperature, relative humidity and light intensity.

## Conclusions

In conclusion, thin, uniform and smooth NiO films were printed successfully using ink-jet printing on ITO coated glass substrates with controlled thickness of as low as 18 nm. Further these thin films were integrated successfully as hole transporting layers in organic solar cell devices. Detailed investigations on various printing parameters showed that good quality NiO films were obtained at a substrate temperature of 25 °C, drop spacing of 50 µm and with substrates UVO treated prior to the printing of NiO films. Further heat treatment temperature of NiO films had profound influence on the device characteristics, governed by decomposition of ink precursor into NiO and other volatile constituents and devices consisting of printed NiO films. Further UV ozone treatment of NiO films resulted in a marked improvement in the performance of the devices which is even slightly better than the performance of the devices fabricated on spin coated NiO films. Although, it is tempting to assign this improvement to the increase in the work function of NiO after surface treatment as studied using KPFM, the underlying reasons appear to be related to the improved quality of interface between the P3HT:PC_60_BM blend and UVO treated NiO film. The organic solar cell devices with device structure ITO/NiO(printed)/P3HT:PC_60_BM/LiF/Al fabricated on printed NiO films (heat treated at 400 °C) and with surface treatment prior to active layer deposition showed a maximum power conversion efficiency of ~2.60%, comparable to that obtained on spin coated NiO films. Stability testing of the NiO devices showed significantly superior atmospheric stability of NiO based devices over PEDOT:PSS based devices.

## Method

First, a NiO precursor solution of concentration 0.44 M was prepared by mixing nickel acetate tetrahydrate as a precursor in 2-methoxy ethanol (C3H8O2) as a solvent with tiny amounts (0.3 ml in 10 ml) of monoethanolamine (C2H7NO) as a stabilizing agent. The mixture was stirred for 12 hour at room temperature (ca. 28 °C). Prior to printing of NiO precursor ink, ITO coated glass substrates were cleaned in soap solution followed by rinsing first in DI water and then ultrasonication in acetone for 15 min followed by 15 minutes in isopropyl alcohol and drying in flowing N_2_ gas. Subsequently, surface of ITO coated glass sample was treated in an UV Ozone cleaner (Model No-42-220, Jelight) for 15 minutes. The NiO thin films were printed on these cleaned substrates using DiMatix2831 inkjet printer with sixteen nozzles with each nozzle having diameter of 21 µm. Based on the approaches used in our previous works^43, 44^, we fixed the jetting voltage at 12 V for printing NiO precursor ink and printing of a uniform NiO film was achieved by optimizing drop spacing and surface treatment of the substrate. Printed NiO films were heat treated in air at 250 °C, 350 °C and 400 °C for 1 hour. Surface uniformity was studied using using optical microscope (Zeiss) while a Dektak surface profilometer was used for thickness measurements. The surface roughness was measured using Asylum Research MFP-3D atomic force microscope. Optical transmittance was measured using UV-Vis spectrophotometer (Perkin Elmer Lambda750). Finally to integrate the printed NiO thin films as hole transporting layers in a organic solar cell devices, the devices with conventional structure (Glass/ITO/NiO/P3HT:PC_60_BM/LiF(1 nm)/Al) were fabricated, where thermally evaporated LiF as used as a electron transport layer while hole transporting layer of NiO was ink-jet printed as well as spin coated (only for control samples). For active layer deposition, the P3HT:PC_60_BM((1:0.8 ratio)) blend solution was prepared in chlorobenzene and followed by stirring on hot plate at 40 °C for 12 hr. Films were spin coated at 1500 rpm for 60 seconds followed by annealing at 150 °C for 10 minutes on a hot plate in the glove box. The top electrodes of Al (100 nm) were thermally evaporated through a shadow mask in a glove-box integrated evaporation chamber at a deposition rate of 0.09 nm/sec while 1 nm thin LiF layer was grown at a rate of 0.01 nm/s. The current voltage characteristics was measured with Keithely 2400 source meter at a scan rate of 0.02Vs^−1^. For measurments under light, a solar simulator (NewportClass ABA Solar Simulators with 2×2 inch^2^ illuminated area) was used with an AM1.5 filter was used having intensity of 100 mWcm^−2^. The light enetred the devices through bottom ITO electrode while illuminated area was only the device pixel area (0.09 cm^2^) and other device area remaining covered, achieved using a shadow mask.

## Electronic supplementary material

Supplementary Information
