# Supplementary material for: Inkjet printing of NiO films and integration as hole transporting layers in polymer solar cells
Source: Sci Rep. 2017 May 11;7:1775. doi: 10.1038/s41598-017-01897-9 (PMC5431859; doi:10.1038/s41598-017-01897-9)
Supplement: Supplementary file 1 — Supplementary Information [file 41598_2017_1897_MOESM1_ESM.pdf]

Supplementary Information

Title: Inkjet printing of NiO films and integration as hole transporting layers in polymer solar cells

Arjun Singh, Shailendra Kumar Gupta, Ashish Garg*

Department of Materials Science and Engineering

Indian Institute of Technology Kanpur, Kanpur-208016, India

***Corresponding authors:** ashishg@iitk.ac.in

Supplementary Figure 1 shows the formation of stable spherical NiO ink drops at a jetting voltage of 12V using ink of surface tension 36.88 dynes/cm, viscosity 2.6cP.

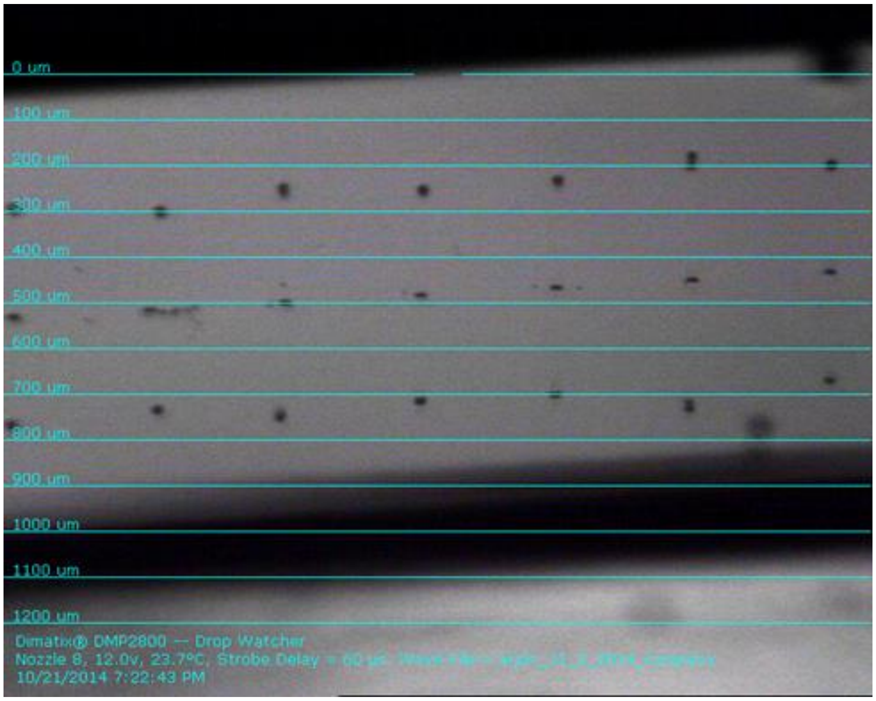

Supplementary Figure 1: Jetting behaviour of NiO precursor ink

Supplementary Figure 2 shows that line width profiles of the printed NiO lines as a function of drop spacing varied from 30 to 60 microns. One can see here that the line width and the edge height both decrease as the drop spacing increases which plays an important role in the formation of a continuous film. The best NiO films were achieved at a drop spacing of 50 µm.

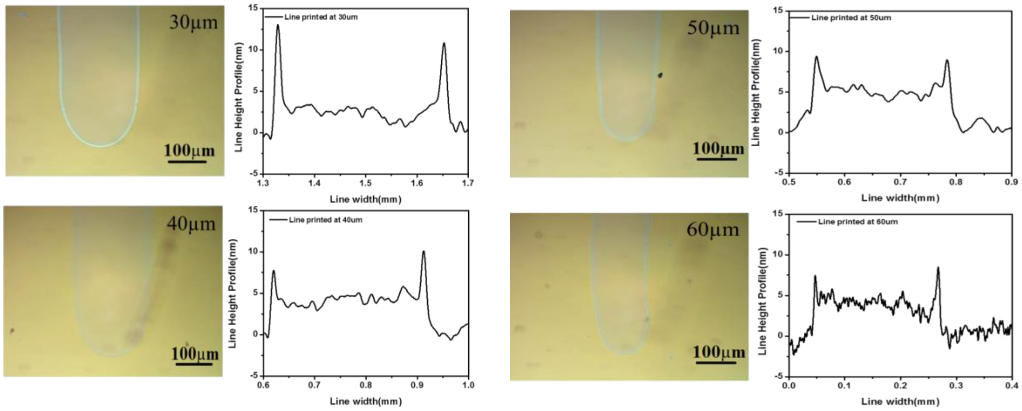

(a)

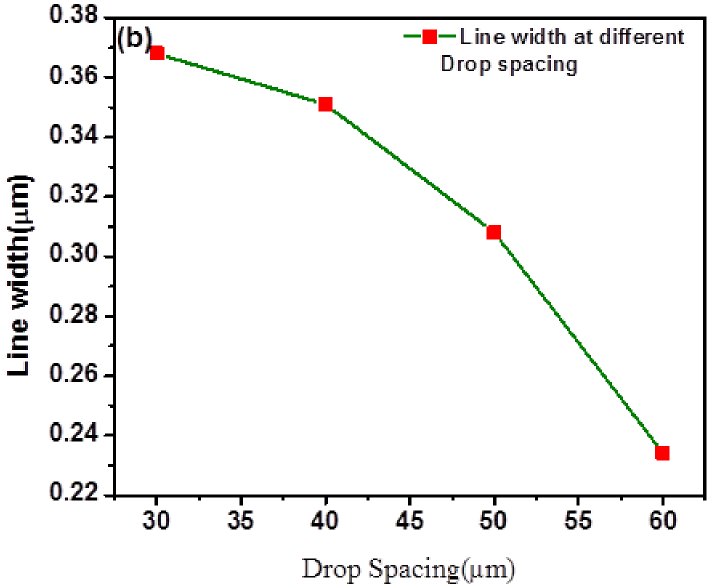

(b)

Supplementary Figure 2: Optical micrographs and 2D line profiles of inkjet printed NiO film at different drop spacing on 15 minutes UVO treated ITO/glass substrates held at 25℃ (b) Line width of inkjet printed NiO film at different drop spacing on 15 minutes UVO treated ITO/glass.

TheSupplementary Figure3 shows the profiles of the lines printed at various substrate temperatures. One can see here that the line width varies linearly with the substrate temperature and the best films with rather smooth edges were achieved at 25ºC.

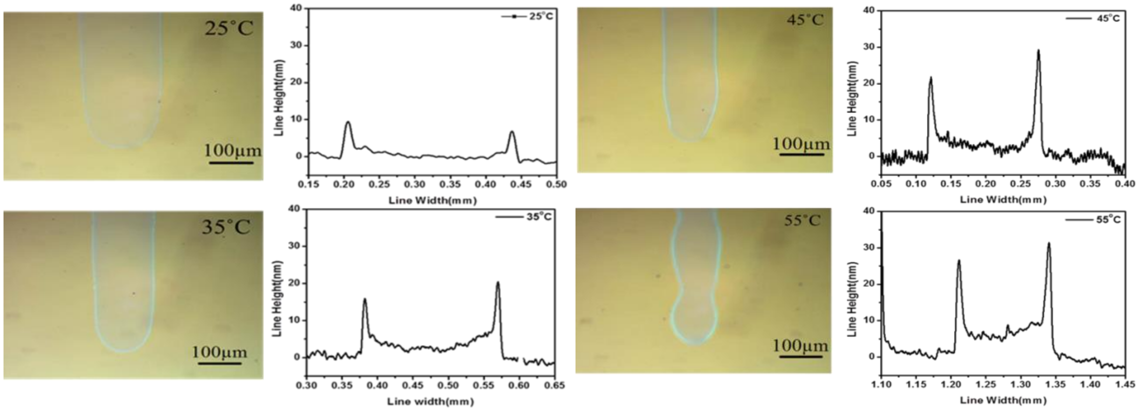

(a)

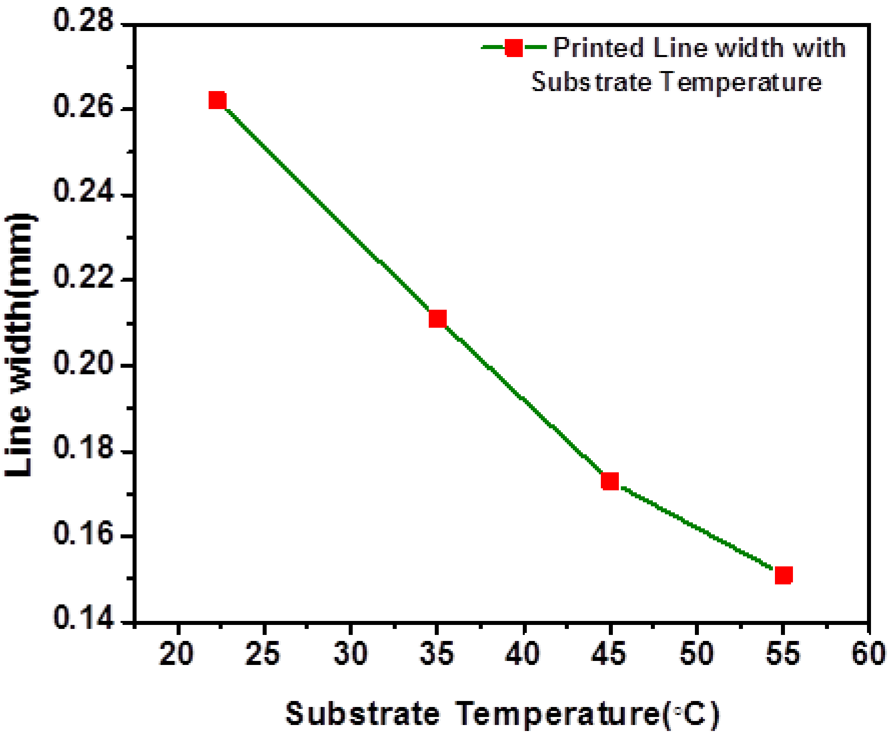

(b)

Supplementary Figure 3: Optical micrographs and 2D printed line profiles of inkjet printed NiO films at different substrates temperature on ITO/glass substrates with UVO surface treatment. Line width of printed line with substrates temperature.
